# Secondary Osteoporosis in Patients with Juvenile Idiopathic Arthritis

**DOI:** 10.4061/2011/569417

**Published:** 2011-02-20

**Authors:** Kristyna Brabnikova Maresova

**Affiliations:** ^1^Institute of Rheumatology, Slupi 4, 128 50, Prague 2, Czech Republic; ^2^Department of Rheumatology, First Faculty of Medicine, Charles University in Prague, Kateřinská 32, 121 08 Prague 2, Czech Republic

## Abstract

Bone disease in patients with juvenile idiopathic arthritis (JIA) is associated with focal (joint erosion and juxtaarticular osteopenia) and systemic bone loss (generalized osteopenia or reduction of bone mass density). Pathophysiology of bone loss is multifactorial and involves particularly proinflammatory cytokines and deleterious effects of glucocorticoid therapy. Clinical studies in patients with JIA indicate excessive activation of osteoclastogenesis and reduction of bone formation. Reduction of physical activity, muscle atrophy caused by high disease activity, and compulsory restriction in movements are also associated with bone loss. In patients with JIA, the disease can be complicated by growth cartilage involvement and systemic or local growth retardation. In the absence of preventive measures, fragility fractures can occur even at an early age.

## 1. Introduction

Juvenile idiopathic arthritis (JIA) is a systemic autoimmune chronic inflammatory joint disease beginning until 16 years of age. JIA is the most frequent rheumatic systemic disease in the childhood. In the industrial countries, the incidence of JIA is 5–18 and prevalence of JIA is 30–150/100 000 children until 16 years old. In the Czech Republic, the annual incidence of JIA is 13/100 000 and prevalence of JIA is 140/100 000 children until 16 years old [1]. The initial cause of the chronic inflammatory processes targeting the synovial lining of joints is not known. Many of the proinflammatory factors stimulate the differentiation of osteoclasts from the hematopoietic precursor. Generalized bone loss of bone mass is a common feature in JIA.

## 2. Low Bone Mass and Increased Risk of Fracture

During childhood and adolescence in patients with JIA, when the peak of bone mass is attained in the healthy people, the accrual of bone mass is suppressed through direct and indirect mechanisms, namely by the inflammatory disease, by drug therapy and immobilization [2, 3]. In JIA, both synovial-derived and soluble cytokines are involved. Osteopenia or osteoporosis occurs in all of the JIA forms, most typically in systemic and polyarticular forms of disease. The low bone mass is associated with the high activity of the disease and with the number of involved joints in JIA patients [4–9], also with the reduction of bone formation [4, 6]. Reduced bone mineral density (BMD) is observed at all sites of the skeleton in the children and adolescents with JIA and also in adults with JIA. In the cross-sectional study, the low BMD in lumber spine and hip was found in 40–52% adult patients with JIA [10]. However, even the full remission of the disease in young adults is not able to completely normalize BMD at all skeletal sites. In 229 young adults with a past history of JIA, persistently low BMD was observed at the femoral neck and on total body [11]. In another study, 41% of adults with a history of JIA had osteopenia [12].

Therapy with glucocorticoids can prevent the acquisition of an optimal peak bone mass in young patients. The lower peak of bone mass is associated with the increased risk of osteoporosis and the increased risk of fracture in the adult age [13–15]. 

**Figure 1:**
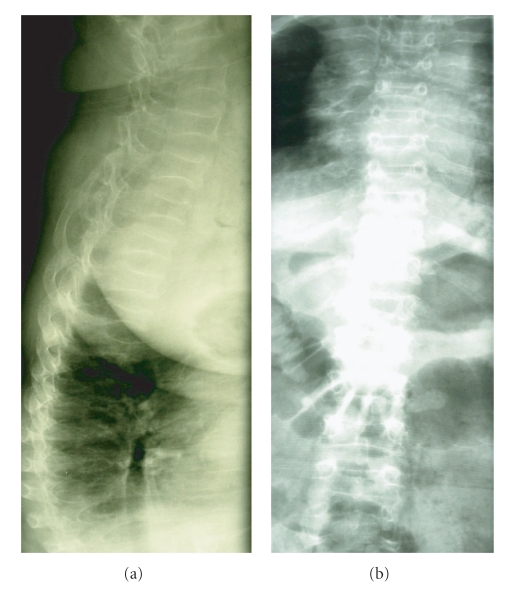
Multiple compressive vertebral fractures and rib fractures in a 22-year-old women diagnosed with JIA at 3 years of age. Bone densitometry (DXA, GE Prodigy): total femur BMD of 0,453 g/cm^2^, T-score −4,5; femur neck BMD of 0,536 g/cm^2^, T-score −4,2.

Drug therapy with glucocorticoids may increase the risk of developing osteopenia and osteoporosis. Vertebral collapse is more common in children receiving a cumulative dose of at least 5 g of prednisone equivalents, prolonged periods of bed rest, and with low BMD and low serum concentrations of 25-hydroxyvitamin D [13, 16]. In a study in 103 patients with JIA, 23% had at least one fracture in the presence of growth failure, articular erosions and high cumulative dose of glucocorticoids; 56% of these fractures were vertebral [17] (Figure 1). 

## 3. Fractures and Low Lean Mass

The increased risk of fracture has been proven especially in patients with erosive arthritis, growth retardation and high cumulative dose of glucocorticoids [2, 3]. 

Myopathy caused by the autoimmune inflammation is one of reasons of the low lean mass in patients with JIA. Proinflammatory cytokines (especially TNF-*α*) stimulate protein degradation, inhibit myocyte differentiation, and cause myocyte apoptosis [18, 19], as demonstrated by the muscle biopsies [20]. Glucocorticoid myopathy can also induce a low lean mass [21]. Patients with JIA are less physically active than healthy population, their physical condition is impaired, and risk of fracture is increased [22, 23]. 

In patients with JIA, there is an important relationship between risk of fracture, bone mineral density and quantity of lean mass. The lean mass corelates with BMD at the various skeletal sites. Bone status in children with JIA (and also in healthy children) markedly depends on the muscle force affecting the skeleton [4, 18]. Reduction of whole body lean mass and higher accrual of fat mass was established at the early phase of the disease [8]. The increased risk of forearm fracture was demonstrated also in healthy children and adolescents with low whole body lean mass and high fat mass [24]. BMD at the cortical and trabecular bone of the forearm and bone and lean muscle geometry was measured in 57 children with JIA using the peripheral quantitative computed tomography (QCT) [18]. Patients with JIA had significantly reduced cross-sectional area of the lean muscle mass (CSA). This reduction notably corelated with lean mucle force and bone geometry abnormalities and with significant reduction of the cortical bone thickness. All of these can be associated with increased risk of fracture. The peripheral QCT was also used to demonstrate a reduced calf lean muscle mass and tibial trabecular BMD and cortical thickness in patients with JIA [25].

## 4. Growth Retardation

The important difference between JIA and rheumatoid arthritis in adults lies in growth retardation of children with JIA, deceleration of growth rate, low stature in adult age and local growth retardation at sites of joints involved in arthritis [2, 3]. A significantly low stature (the final height below −2 standard deviation, SD) was demonstrated in 11% of children with JIA, and in 41% children with systemic form of disease [26]. Sources of growth retardation in JIA are multifactorial; chronic inflammation and long-term glucocorticoid treatment are the most relevant. The linear growth can be improved with remission of the disease. 

The growth hormone (GH) and insulin-like growth factor I (IGF-I) are the most important regulators of postnatal growth. In children with JIA and serious growth retardation, the normal pulsative secretion of growth hormone, but low levels of IGF-I were described (resistance to growth hormone). Proinflammatory cytokines influence linear growth of children by their systemic effects and by local effects on growth cartilage of long bones [26]. Increased production of interleukin 6 (IL-6) and interleukin 1 beta (IL-1*β*) accelerates degradation of insulin-like growth factor I binding protein 3 (IGFBP-3) resulting in reduction of IGF-I levels and growth retardation. Chondrocyte apoptosis induced by the tumor necrosis factor *α* (TNF*α*) through the medium Fas-associated death domain (FADD) acts the important part in growth abnormalities [26]. Growth retardation caused by chronic inflammation and glucocorticoid treatment can be positively influenced by growth hormone treatment [27].

## 5. Biochemical Markers of Bone Turnover

Studies of bone formation and bone resorption markers are not unambiguous; however, most studies indicated prevalence of bone resorption over bone formation [5], the others conversely indicate reduction of the bone formation [4, 6, 28]. Decreased bone formation in the course of adolescent growth spurt obstructs achievement of peak of bone mass and increase risk of fracture in the adult age [4, 13, 14]. Successful treatment of the disease is associated with elevation of bone formation markers [6].

In prepubertal children with active JIA, prevalence of bone resorption markers over reduced markers of bone formation corelated with laboratory indices of disease activity, namely in children with polyarticular phase of JIA [4, 6, 28, 29]. Except for the high disease activity, reduced concentrations of bone turnover markers and low BMD were found in patients with joint destruction and longer disease duration [29]. According to Pereira et al., bone formation in patients with JIA was suppressed from early to middle puberty, while at older patients with JIA, the main factor of bone loss was the elevation of bone resorption [30]. Thus, similarly to adults with rheumatoid arthritis, the proinflammatory cytokines (TNF-*α*, IL-1) produced by synovial membrane are responsible for excessive bone resorption also in adult patients with JIA [31].

## 6. Cellular and Molecular Mechanisms of Bone Remodeling in Patients with JIA

### 6.1. Influence of Proinflammatory Cytokines on Bone Remodeling

Proinflammatory cytokines, such as tumor necrosis factor *α* (TNF*α*), interleukin 1 (IL-1), interleukin 6 (IL-6) a interleukin 17 (IL-17), present in arthritic joint can cause an excessive osteoclastogenesis [32–35]. TNF*α* significantly elevates bone resorption [36] and attenuates osteoblastogenesis and bone formation [37–40]. IL-1 also accelerates osteoclast maturation [41]. In patients with rheumatoid arthritis, treatment with TNF*α* antibodies demonstrably reduces joint erosions [32]. IL-17, which is produced by T-lymfocytes [34, 42, 43] induces osteoclast differentiation by increasing expression of RANKL and RANK. On the contrary, IL-17 suppresses expression of osteoprotegerin in osteoblasts resulting in prevalence of bone resorption over bone formation and bone loss [6, 34, 44–46].

### 6.2. RANKL/RANK/OPG Triad in Patients with JIA

Receptor activator nuclear factor kappa B (RANK) and its ligand (*Ŕ*ANKL) have essential importance for osteoclastogenesis and osteoclast function [47–50]. Osteoprotegerin (OPG) is a soluble decoy receptor for RANKL produced by osteoblasts. OPG binding to RANKL prevents RANKL activation of RANK and thus activation of the osteoclastogenesis [48, 51]. High RANKL/OPG ratio results in prevalence of bone resorption over bone formation [52].

Increased production of RANKL in synovial fluid and increased concentrations of RANKL in serum are found in adults with rheumatoid arthritis [53]. An increased RANKL/OPG ratio was observed in children with juvenile dermatomyositis, and in patients with juvenile idiopathic arthritis [54, 55]. In the later study in patients with JIA, increased serum OPG concentrations were not sufficient to compensate for increased levels of RANKL [54, 55]. Serum concentrations of RANKL were increased in all the forms of JIA [55]. An increased RANKL/OPG ratio was also found in a subset of 30 girls with polyarticular course of highly active JIA and joint erosions [56], and in synovia in patients with polyarticular and enthesitis-related forms of JIA [57].

### 6.3. Wingless Proteins (Wnt) Signalling Pathway

The extent of the bone involvement in the rheumatic inflammatory processes depended on age and bone maturity [34]. In children with JIA, decreased osteoblastic formation and function may contribute to the bone loss [30, 34, 58, 59]. With this respect, the proinflammatory cytokines (especially TNF*α*), stimulate production of inhibitors of the Wnt proteins signalling pathway, especially sclerostin and Dickkopf 1 (DKK-1) and consequently inhibit osteoblast differentiation [60–63] (Figure 1). 

The Wnt proteins are large family of factors that bind to cell-surface receptors of the Frizzled family, causing the receptors to activate Dishevelled family proteins and ultimately resulting in a change in the amount of *β*-catenin that reaches the nucleus and interacts with T-cell factor/lymhoid enhancer factor (TCF/LEF) family transcription factors to promote specific gene expression [64]. Decreased production of DKK-1, an inhibitor of the Wnt signaling pathway, is associated with reverse of osteoresorptive pattern in mouse model of rheumatoid arthritis to pattern of osteoarthritis with increased bone formation and osteophyte formation [61]. The DKK-1 blockade is associated with stimulation of OPG production by osteoblasts and consequent decrease in bone resorption [61, 64]. The importance of Wnt proteins for susceptibility to JIA was confirmed in the study of Wnt-1 inducible signaling pathway protein 3 (WISP3) polymorphism [65].

### 6.4. Matrix Metalloproteinases and Their Inhibitors in Patients with JIA

Matrix metalloproteinases (MMPs) are responsible for cartilage destruction and periarticular bone erosions in juvenile idiopathic arthritis [21, 55, 66–68]. MMP1/tissue inhibitor of metalloproteinases 1 (TIMP1) and MMP3/TIMP1 ratios are significantly higher in all the forms of JIA in comparison to healthy controls. These ratios significantly correlate with disease activity and could be efficacious biomarkers for monitoring of disease development [55].

## 7. Effects of Glucocorticoids, and Disease-Modifying Antirheumatic Drugs (DMARDs)

In children, a negative correlation between bone mass and cumulative dose of glucocorticoids was found [69–71]. During the childhood and adolescence, glucocorticoids can impair physiological process of bone mass accumulation and can cause deterioration of peak of bone mass resulting in increased risk of fracture in future life. 

Methotrexate, which is the most frequent DMARDs in children, can cause osteopenia in children patients with malignancies, but low-dose methotrexate used in inflammatory diseases did not influence negatively the bone mass [72, 73].

## 8. Therapeutic Options to Improve Bone Status in JIA

### 8.1. TNF*α* Antibodies Treatments

Biological treatment with infliximab and etanercept in children with JIA is associated with decrease in disease activity [74–76]. The positive influence of treatment with TNF*α* antibodies was also documented upon the skeleton. Simonini was the first to demostrate increased bone mass after 1-year etanercept treatment in children with JIA; reduction of bone loss was associated with therapeutic response with decreased disease activity [75]. Etanercept also improves the linear growth in children with JIA [76].

### 8.2. Antiresorption Treatments

Aminobisphosphonates represent an effective option in patients with documented prevalence of bone resorption over bone formation [77]. In JIA, calcium and vitamin D, calcitonin, and aminobisphosphonates have been studied [78–80]. However, studies treated small numbers of patients with different characteristics of JIA, and controlled studies on both preventive strategies and treatment in augmenting bone mass and reducing the fracture risk are still lacking.

### 8.3. Bone Anabolic Treatments

Growth hormone was effective in stimulating collagen production and improving liner growth in JIA [81–83]. However, long-term controlled studies are needed to determine impact on bone mass and bone turnover and the risks of growth hormone therapy [84]. 

In adult patients with closed linear growth and severe osteoporosis, an intermittent administration of PTH 1–34 (teriparatide) or PTH 1–84 represents an effective option to restore bone structure that has previously been lost [85].

## 9. Conclusion

Well-timed and efficient treatment of JIA in children and adolescents can improve bone status. However, patients who suffered from JIA during childhood and adolescence may attain decreased bone mass and have an increased risk of fragility fractures. It is important to identify the subjects with an increased risk of fracture as early as possible. In adult patients with closed linear growth, it is necessary not only to reduce bone resorption, but also to support formation of new healthy bone mass. Several new therapeutical procedures are under investigation.
